# The TyG Index and Obesity Indicators Predicting Low Muscle Mass in US Adults Without Diabetes: NHANES 2011–2018

**DOI:** 10.1155/jnme/1195681

**Published:** 2026-07-13

**Authors:** Jiaqi Huo, Fang Li, Ting Wang, Simiao Tang, Yue Xi, Genqing Xie

**Affiliations:** ^1^ Department of Clinical Nutrition, The First People’s Hospital of Xiangtan City, Xiangtan, 411100, Hunan, China; ^2^ Hunan Center for Evidence-Based Medicine, Clinical Epidemiology Laboratory, the Second Xiangya Hospital, Central South University, Changsha, 410011, Hunan, China, csu.edu.cn; ^3^ Department of Epidemiology, School of Public Health, Sun Yat-Sen University, Guangzhou, 510275, Guangdong, China, sysu.edu.cn

**Keywords:** insulin resistance, low muscle mass, obesity, TyG index

## Abstract

**Background:**

Insulin resistance and obesity are critical determinants of low muscle mass. The main goal was to determine whether the reduced muscle mass in people without diabetes is correlated with the insulin resistance index and obesity indicators.

**Method:**

The data from NHANES 2011 to 2018 were analyzed to explore how the TyG index and obesity indices relate to low muscle mass. Associations were conducted using weighted logistic regression, RCS analysis, sensitivity analyses, and subgroup evaluations. In addition, the prediction capability of these indices was evaluated using ROC curve analysis and the AUC.

**Result:**

The TyG index (OR = 1.86, 95% CI: 1.31–2.63, *p* = 0.001), TyG‐WC (OR = 7.53, 95% CI: 5.49–10.30, *p* < 0.001), TyG‐WHtR (OR = 10.40, 95% CI: 7.56–14.30, *p* < 0.001), and TyG‐WWI (OR = 4.74, 95% CI: 3.41–6.57, *p* < 0.001) turned out to show a positive relationship with low muscle mass in nondiabetic adults. TyG‐WHtR’s higher discriminative performance has been proven by its AUC, which was 0.831 (95% CI: 0.814–0.848). The most appropriate threshold values were 4.932 for men and 5.031 for women aged ≥ 40 years, and 4.628 for men and 4.674 for women aged < 40 years.

**Conclusion:**

The risk of insufficient muscle mass is increased in nondiabetic adults with higher values of TyG‐obesity indicators (TyG‐WC, TyG‐WHtR, and TyG‐WWI). TyG‐WHtR possessed the best discriminative ability than other indicators.

## 1. Introduction

Low muscle mass is the core indicator of sarcopenia. The methodologies such as BIA, DEXA, CT, and MRI [1, 2], which are commonly used to assess muscle mass, present significant practical limitations, particularly in terms of accessibility and feasibility for routine use in primary care environments. As a result, there is a growing imperative to investigate alternative strategies for assessing low muscle mass to identify this condition at an early stage.

Insulin resistance (IR) is acknowledged as a key pathophysiological driver in low muscle mass development. In older people without diabetes who live in the community, Lee et al. demonstrated that IR is a reliable indicator of reduced muscle mass [3]. In a cohort encompassing both young and older apparently adults, Aleman‐Mateo et al. found IR to be strongly correlated with the reduction in relative appendicular skeletal muscle mass (ASM) [4]. Collectively, these findings strengthen the case for incorporating IR into assessment frameworks for muscle mass loss.

Triglycerides and fasting blood glucose are two routinely measured metabolic parameters that are used to calculate the TyG index, which has been shown to be a reliable indicator for measuring IR [5, 6]. Emerging evidence shows that a greater TyG index has been implicated in both a higher probability of muscle mass loss and an accelerated depletion of lean mass, positioning it as a potential prognostic indicator [6–9]. Furthermore, the predictive accuracy of IR can be improved by incorporating anthropometric adiposity measures with the TyG index, as proposed by several investigations [10]. However, to date, most investigations employing the TyG‐obesity composite indices have focused primarily on cardiovascular disease [11–13] and NAFLD [14–16]. However, the application of TyG‐obesity indices to the outcome of low muscle mass remains largely unexplored.

Given the potential confounding effect of hypoglycemic agents on diabetic patients’ fasting glycemia, the current investigation examined how the TyG‐obesity composite indices correlated with low muscle mass exclusively in the nondiabetic population. The goal was to provide novel insights and evidence that could facilitate the identification of low muscle mass.

## 2. Methods

### 2.1. Research Subjects and Design

**FIGURE 1:**
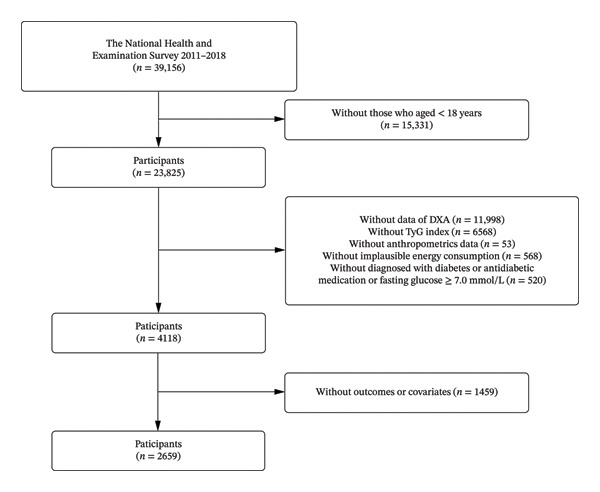
The workflow for participants’ enrollment.

In the end, 2659 adults were ultimately included in this cross‐sectional survey, whose data were used from NHANES 2011–2018. Figure 1 illustrates the workflow for participants’ enrollment. The participants meeting any of the ensuring requirements were excluded: participants under 18 years of age and those with an ADA‐defined diagnosis of diabetes [16]; incomplete data for DEXA measurements, TyG index, or TyG‐obesity composites (BMI, WC, WHtR, and WWI); daily energy consumption outside the plausible range (800–4200 kcal for man; 500–3500 kcal for women) [17]; and missing covariate data. All subjects provided informed consent in writing after the NCHS Research Ethics Review Board gave their approval.

### 2.2. Definition of TyG, TyG‐WC, TyG‐WHtR, and TyG‐WWI Indices

During the mobile examination, the researchers measured the FBG together with triglyceride levels from the biological specimens provided by the participants and then calculated the TyG index. Simultaneously, anthropometric parameters were obtained through standardized physical examinations. From these measurements, the WHtR and WWI were subsequently derived. For analytical purposes, subjects were divided into two groups using each index’s median cutoff, setting the lowest quartile (Q1) as the comparator.

**FIGURE 2:**
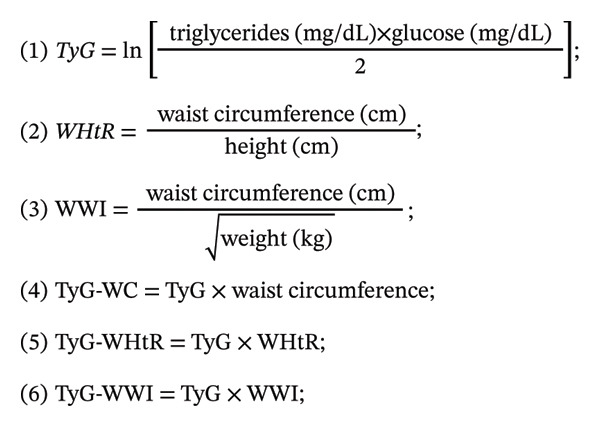
The calculation of TyG‐obesity composite indicators.

The calculation methods for TyG‐obesity composite indices are shown in Figure 2.

### 2.3. Definition of Low Muscle Mass

Body weight–indexed appendicular lean mass (ALM)/weight was used as the primary measure of relative muscle mass. A value below the 25th percentile (<*P*_25_) of the study population was considered indicative of low muscle mass [18]. Composition of the body was performed by DEXA (QDR‐4500A, Hologic Inc., Bedford, MA, USA), and ALM was derived by processing total‐body scans data with Hologic APEX software (Version 4.0; Hologic Inc.). Consistent with DEXA protocols, participants were excluded if they were pregnant, weighed > 136 kg, or had a height > 196 cm.

### 2.4. Assessment of Covariates

A comprehensive set of covariates was included based on previously established associations with muscle mass. Covariates comprised age, gender, BMI, level of education, marital status [19], PIR, physical exertion, smoking status, alcohol use, cancer, hypertension, and dietary intake (energy, protein, and vitamin D).

Physical exercise data were derived from the physical activity questionnaire. MET scores were calculated from reported activity frequency and duration using standard MET values for each activity type [20]. Accordingly, participants were grouped by physical activity level: sedentary (< 1 MET‐h/week), moderately active (1–48 MET‐h/week), and highly active (> 48 MET‐h/week). The PIR was also categorized as < 1.30, 1.30–3.50, and ≥ 3.50 [21].

Smoking and alcohol use were evaluated through the application of the following two questions: “Have you had 12 or more alcoholic beverages annually?” and “Have you smoked 100 or more cigarettes over your lifetime?” Cancer and hypertension status were identified via self‐reported physician‐diagnosed history.

### 2.5. Statistical Analysis

All analyses were conducted using R (Version 4.3.1, R Foundation for Statistical Computing, Vienna, Austria). Categorical variables were reported as N (%) and continuous variables as mean ± SD. Group comparisons were assessed via the chi‐square test for categorical data and ANOVA for continuous data. The connections of TyG, TyG‐WC, TyG‐WHtR, and TyG‐WWI with low muscle mass were evaluated using weighted multivariable logistic regression, reporting ORs (95% CIs). We performed a sensitivity analysis following exclusion of those on lipid‐lowering drugs to test result’s robustness. To study whether there are probable nonlinear relations linking the TyG index (and its combinations with obesity indices) to low muscle mass, we used restricted cubic spline (RCS) regression. ROC curve analysis, with corresponding AUC values, was applied to evaluate the TyG, TyG‐WHtR, TyG‐WWI, and TyG‐WC’s performance in identifying low muscle mass. The ROC curve comparisons were conducted via the DeLong test with Bonferroni correction. A two‐tailed Type I error rate of 0.05 was applied (*α* = 0.05).

## 3. Results

### 3.1. Baseline Features of the Study Population

The demographic and clinical features of the 2659 non‐diabetic adults from NHANES 2011–2018 are summarized in Table 1. The participants’ mean age was 37.9 ± 11.5 years, and the cohort comprised 50.4% females and 48.1% married individuals.

**TABLE 1:** Baseline features of study subjects (*n* = 2659, *n* (%)).

Characteristics	Overall *n* = 2659	Low muscle mass		*p*
		Yes *n* = 665 (25.0)	No *n* = 1994 (75.0)	
Age, years	37.9 ± 11.5	41.3 ± 11.3	36.8 ± 11.3	**< 0.001**
BMI, kg/m^2^	27.9 ± 6.08	32.7 ± 6.05	26.4 ± 5.19	**< 0.001**
Gender, *n* (%)				1.000
Female	1339 (50.4)	335 (50.4)	1004 (50.4)	
Male	1320 (49.6)	330 (49.6)	990 (49.6)	
Education level, *n* (%)				**0.006**
Middle school or below	431 (16.2)	135 (20.3)	296 (14.8)	
High school	558 (21.0)	136 (20.5)	422 (21.2)	
College graduate or above	1670 (62.8)	394 (59.2)	1276 (64.0)	
Marriage status, *n* (%)				**< 0.001**
Married	1278 (48.1)	352 (52.9)	926 (46.4)	
Never married	739 (27.8)	133 (20.0)	606 (30.4)	
Other	642 (24.1)	180 (27.1)	462 (23.2)	
PIR, *n* (%)				**0.007**
< 1.30	865 (32.5)	248 (37.3)	617 (30.9)	
1.30–3.50	929 (34.9)	220 (33.1)	709 (35.6)	
≥ 3.50	865 (32.5)	197 (29.6)	668 (33.5)	
Physical activity level, *n* (%)				**< 0.001**
Sedentary	453 (17.0)	162 (24.4)	291 (14.6)	
Moderately active	1182 (44.5)	276 (41.5)	906 (45.4)	
Highly active	1024 (38.5)	227 (34.1)	797 (40.0)	
Smoke, *n* (%)				**0.002**
Yes	1047 (39.4)	304 (45.7)	743 (37.3)	
No	1612 (60.6)	361 (54.3)	1251 (62.7)	
Alcohol use, *n* (%)				0.768
Yes	2055 (77.3)	511 (76.8)	1544 (77.4)	
No	604 (22.7)	154 (23.2)	450 (22.6)	
Self‐reported cancer, *n* (%)				**0.015**
Yes	91 (3.4)	34 (5.1)	57 (2.9)	
No	2568 (96.6)	631 (94.9)	1937 (97.1)	
Hypertension, *n* (%)				**< 0.001**
Yes	520 (19.6)	189 (28.4)	331 (16.6)	
No	2139 (80.4)	476 (71.6)	1663 (83.4)	
Dietary energy, kcal/d	2070.00 ± 701.00	2010.00 ± 702.00	2100.00 ± 700.00	**0.004**
Dietary protein, gm/d	83.10 ± 34.70	78.80 ± 32.40	84.50 ± 35.30	**< 0.001**
Dietary vitamin D, mcg/d	4.51 ± 4.55	4.03 ± 3.95	4.67 ± 4.72	**0.006**
TyG index	8.41 ± 0.62	8.64 ± 0.58	8.34 ± 0.61	**< 0.001**
TyG‐WC	804.00 ± 158.00	936.00 ± 149.00	760.00 ± 135.00	**< 0.001**
TyG‐WHtR	4.78 ± 0.94	5.61 ± 0.85	4.51 ± 0.79	**< 0.001**
TyG‐WWI	90.30 ± 10.30	98.00 ± 8.92	87.80 ± 9.45	**< 0.001**

*Note:* Bold indicates statistically significant values, *p* < 0.05.

When compared with normal‐muscle individuals, their low‐muscle counterparts exhibited a higher mean age; lower education levels; reduced annual family income; decreased physical activity; and lower dietary intakes of protein, energy, and vitamin D. In addition, participants with low muscle mass exhibited a higher TyG index (8.64 ± 0.58 vs. 8.34 ± 0.61, *p* < 0.001) and higher values of TyG‐composite obesity indices: TyG‐BMI (283.00 ± 55.90 vs. 220.00 ± 49.40, *p* < 0.001), TyG‐WC (936.00 ± 149.00 vs. 760.00 ± 135.00, *p* < 0.001), TyG‐WHtR (5.61 ± 0.85 vs. 4.51 ± 0.79, *p* < 0.001), and TyG‐WWI (98.00 ± 8.92 vs. 87.80 ± 9.45, *p* < 0.001). Furthermore, low muscle mass correlated with an increased probability of smoking, tumor history, and hypertension.

### 3.2. Association Between TyG, TyG‐WC, TyG‐WHtR, and TyG‐WWI With Low Muscle Mass

Presented in Table 2 are the associations linking the TyG index and obesity indices jointly to low muscle mass. Significant positive associations were found for every combined TyG‐obesity indicator in the unadjusted model. The ORs for the TyG index, TyG‐WC, TyG‐WHtR, TyG‐WWI were 2.32 (95% CI: 1.71–3.15, *p* = 0.001), 8.69 (95% CI: 6.27–12.00, *p* < 0.001), 12.00 (95% CI: 8.69–16.60, *p* < 0.001) and 5.90 (95% CI: 4.31–8.08, *p* < 0.001), respectively. The correlations between low muscle mass and TyG index (OR = 1.86, 95% CI: 1.31–2.63, *p* = 0.001), TyG‐WC (OR = 7.53, 95% CI: 5.49–10.30, *p* < 0.001), TyG‐WHtR (OR = 10.40, 95% CI: 7.56–14.30, *p* < 0.001), and TyG‐WWI (OR = 4.74, 95% CI: 3.41–6.57, *p* < 0.001) remained statistically significant after controlling for pertinent factors. Sensitivity analysis results are shown in Appendix Table S2. In sensitivity analysis excluding individuals on lipid‐lowering therapy, the TyG‐obesity indices remained significantly associated with low muscle mass. Significant linear trends were confirmed regarding the links of TyG‐obesity indices to low muscle mass. In the completely adjusted model, the combined indices showed notable linear trends, especially TyG‐WHtR, TyG‐WWI, and TyG‐WC (all *p* for trend < 0.001).

**TABLE 2:** Association between low muscle mass and the TyG index, TyG‐WC, TyG‐WHtR, and TyG‐WWI in US adults in NHANES 2011–2018 (OR [95% CI], *n* = 2659).

	Crude model		Model I		Model II		Model III	
	Crude OR (95% CI)	*p*	Adjusted OR (95% CI)	*p*	Adjusted OR (95% CI)	*p*	Adjusted OR (95% CI)	*p*
TyG index	2.29 (1.86, 2.81)	**< 0.001**	2.00 (1.59, 2.53)	**< 0.001**	1.98 (1.56, 2.51)	**< 0.001**	1.88 (1.47, 2.40)	**< 0.001**
Q1	Reference		Reference		Reference		Reference	
Q2	2.32 (1.71, 3.15)	**< 0.001**	1.95 (1.41, 2.70)	**< 0.001**	1.91 (1.37, 2.66)	**< 0.001**	1.86 (1.31, 2.63)	**0.001**
*p* for trend	**< 0.001**		**< 0.001**		**< 0.001**		**0.001**	
TyG‐WC	4.19 (3.56, 4.94)	**< 0.001**	4.13 (3.53, 4.83)	**< 0.001**	4.18 (3.56, 4.91)	**< 0.001**	4.11 (3.48, 4.86)	**< 0.001**
Q1	Reference		Reference		Reference		Reference	
Q2	8.69 (6.27, 12.00)	**< 0.001**	8.00 (5.84, 11.00)	**< 0.001**	8.00 (5.87, 10.90)	**< 0.001**	7.53 (5.49, 10.30)	**< 0.001**
*p* for trend	**< 0.001**		**< 0.001**		**< 0.001**		**< 0.001**	
TyG‐WHtR	4.57 (3.87, 5.39)	**< 0.001**	4.58 (3.88, 5.42)	**< 0.001**	4.64 (3.91, 5.51)	**< 0.001**	4.59 (3.85, 5.48)	**< 0.001**
Q1	Reference		Reference		Reference		Reference	
Q2	12.0 (8.69, 16.60)	**< 0.001**	10.90 (7.90, 15.20)	**< 0.001**	10.90 (7.98, 15.00)	**< 0.001**	10.40 (7.56, 14.30)	**< 0.001**
*p* for trend	**< 0.001**		**< 0.001**		**< 0.001**		**< 0.001**	
TyG‐WWI	3.42 (2.87, 4.08)	**< 0.001**	3.29 (2.77, 3.90)	**< 0.001**	3.26 (2.75, 3.87)	**< 0.001**	3.19 (2.68, 3.79)	**< 0.001**
Q1	Reference		Reference		Reference		Reference	
Q2	5.90 (4.31, 8.08)	**< 0.001**	5.14 (3.74, 7.06)	**< 0.001**	5.04 (3.67, 6.91)	**< 0.001**	4.74 (3.41, 6.57)	**< 0.001**
*p* for trend	**< 0.001**		**< 0.001**		**< 0.001**		**< 0.001**	

*Note:* The crude model was unadjusted. Model I was adjusted for age, sex, education, marriage status, and PIR. Model II was adjusted for Model I + dietary energy, protein, and vitamin D. Model III was adjusted for Model II + smoking, alcohol use, exercise, self‐reported cancer, and high blood pressure. Bold indicates statistically significant values.

### 3.3. RCS Regression of TyG, TyG‐WC, TyG‐WHtR, and TyG‐WWI in Relation to Low Muscle Mass

**FIGURE 3:**
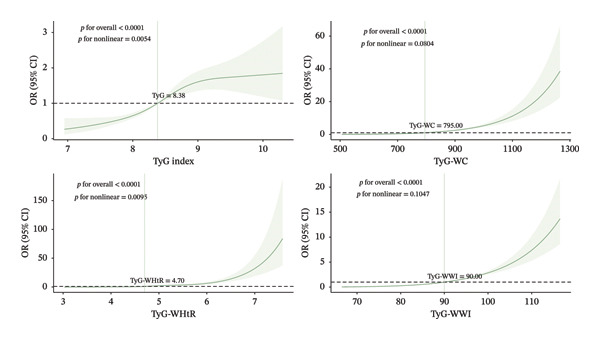
The correlation between TyG, TyG‐WC, TyG‐WHtR, and TyG‐WWI with low muscle mass by RCS regression analysis.

RCS curves illustrating the relationships between low muscle mass and TyG‐obesity indices are shown in Figure 3. After full adjustment in Model III, linear associations with low muscle mass were found for TyG‐WC and TyG‐WWI (*p*‐overall < 0.0001, *p*‐nonlinear > 0.05), whereas TyG index alone and TyG‐WHtR displayed nonlinear associations (*p*‐overall < 0.0001, *p*‐nonlinear < 0.05). Specifically, a positive dose–effect relationship emerged when TyG‐WHtR surpassed 4.70, with higher values of this combined index conferring a progressively elevated risk for low muscle mass.

### 3.4. ROC Curves of TyG, TyG‐WC, TyG‐WHtR, and TyG‐WWI in Relation to Low Muscle Mass

**FIGURE 4:**
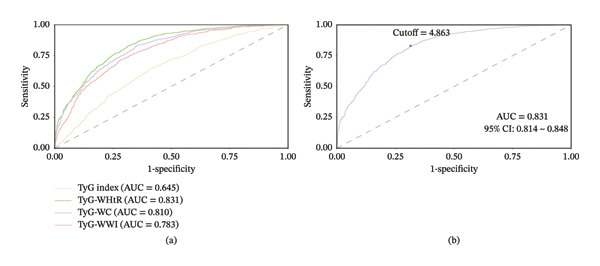
(a) ROC curves of TyG, TyG‐BMI, TyG‐WHtR, TyG‐WC, and TyG‐WWI in relation to low muscle mass. (b) Description of ROC curves of TyG‐WHtR.

Figure 4a shows the discriminative ability of the TyG‐composite obesity indicators for identifying low muscle mass. Having an AUC of 0.831 (95% CI: 0.814–0.848), TyG‐WHtR possessed the greatest diagnostic performance of all indices. In comparison, the values of AUC were 0.810 (95% CI: 0.792–0.828) for TyG‐WC, 0.783 (95% CI: 0.764–0.802) for TyG‐WWI, and 0.645 (95% CI: 0.622–0.669) for TyG index alone. The optimal cutoff value of TyG‐WHtR for predicting low muscle mass was 4.863, with a specificity of 0.685 and a sensitivity of 0.830 (see Figure 4b).

### 3.5. Association Between TyG‐WHtR and Low Muscle Mass in Different Subgroups

**FIGURE 5:**
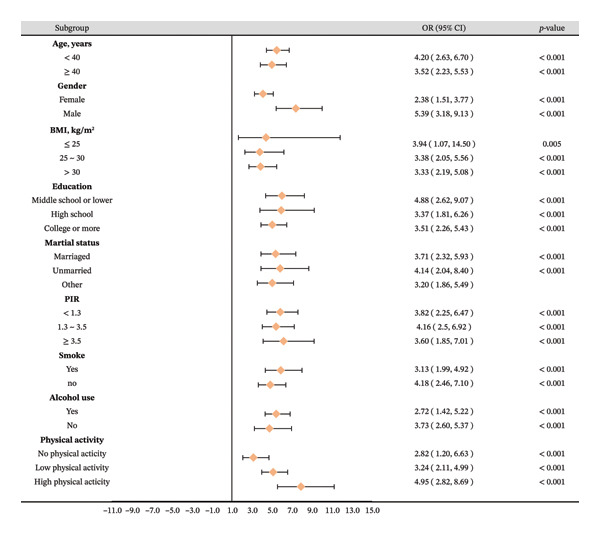
The results of stratified analysis in NHANES 2011–2018.

How TyG‐WHtR positively relates to low muscle mass persisted across all demographic and clinical subgroups in stratified analyses, mirroring the findings from the main analysis. The summary findings of these analyses are depicted in Figure 5.

### 3.6. The Diagnostic Efficacy Between Low Muscle Mass and TyG‐WHtR in Sex‐ and Age‐Stratified Subgroups

**FIGURE 6:**
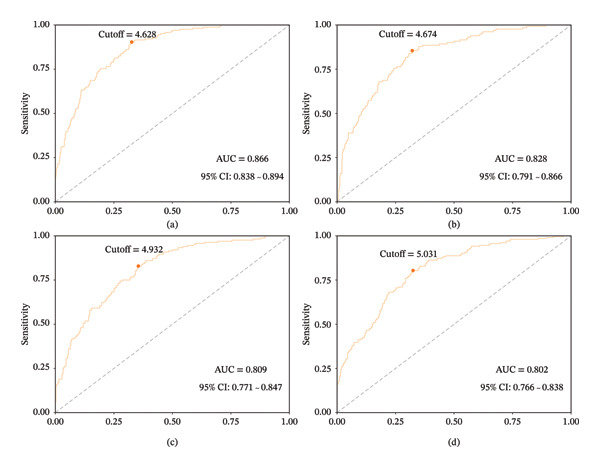
Description of ROC curves of TyG‐WHtR in sex‐ and age‐stratified subgroups.

Figure 6 demonstrates that the ideal TyG‐WHtR cutoff values for diagnosing low muscle mass differ by age and gender. In the population aged ≥ 40 years, the optimal cutoff values were 4.932 (AUC = 0.809, 95% CI: 0.771–0.847) for males and 5.031 (AUC = 0.802, 95% CI: 0.766–0.838) for females, respectively. In contrast, in the population aged < 40 years, the corresponding optimal cutoff values were 4.628 (AUC = 0.866, 95% CI: 0.838–0.894) for males and 4.674 (AUC = 0.828, 95% CI: 0.791–0.866) for females. Table 3 compares the diagnostic efficacy of subgroup‐derived versus universal cutoff values. Comparative analysis of stratified and overall ROC curves indicated that the diagnostic performance of subgroup‐specific cutoff values did not differ significantly from the universal cutoff (≥ 40 and female vs. general *p* = 0.159, ≥ 40 and male vs. general *p* = 0.310, < 40 and female vs. general *p* = 0.903, < 40 and male vs. general *p* = 0.040).

**TABLE 3:** Comparison of diagnostic efficacy of TyG‐WHtR in sex‐ and age‐stratified subgroups and universal population (*n* = 2659).

Subgroups	Stratified				Universal				*p*
	Cutoff	Sensitivity	Specificity	Youden	Cutoff	Sensitivity	Specificity	Youden	
≥ 40 and female	5.031	0.804	0.675	0.479	4.863	0.863	0.605	0.468	0.159
≥ 40 and male	4.932	0.829	0.646	0.475	4.863	0.854	0.613	0.467	0.310
< 40 and female	4.674	0.855	0.679	0.534	4.863	0.802	0.713	0.515	0.903
< 40 and male	4.628	0.904	0.674	0.578	4.863	0.789	0.761	0.550	0.040

## 4. Discussion

This study provides the first evidence, to our knowledge, linking the TyG composite obesity indices, particularly the recently developed WWI, to low muscle mass among individuals without diabetes. Focusing on a nondiabetic population minimized potential confounding from hypoglycemic agents and diabetes‐related complications, which may independently influence muscle mass. This approach enables a clearer understanding of the relationship between IR and muscle depletion, while also providing preliminary evidence to inform muscle‐targeted strategies for the primary prevention of diabetes. According to our research, reduced muscle mass risk is linked to increasing TyG index, TyG‐WHtR, TyG‐WWI, and TyG‐WC. TyG‐WHtR exhibited superior diagnostic ability than other TyG‐obesity composite indices in detecting low muscle mass. The superior performance of TyG‐WHtR supports its potential as an easily derivable screening index for low muscle mass, particularly in clinical settings.

### 4.1. How TyG Index Relates to Low Muscle Mass

Echoing earlier findings, we noted that a TyG index higher than 8.38 was linked to higher probabilities of low muscle mass among nondiabetic adults [7, 18, 22]. The TyG index, a well‐validated proxy for IR [6], is recognized as a contributing factor to the depletion of muscle mass [23]. Moon [24] and Izzo et al. [25, 26] suggested that low muscle mass is linked to IR, and that Type 2 diabetes mellitus increases the risk of sarcopenia, respectively. When IR occurs, featuring “anabolic resistance” [27] in skeletal mass, the suppression of insulin‐dependent anabolic signaling, including the mammalian target of rapamycin (mTOR) pathway, takes place. This suppression attenuates mRNA translation [28, 29], which hinders the synthesis of muscle protein and increases net muscle atrophy. The contribution of IR to muscle depletion is further supported by Wan et al. [30], who demonstrated in a Chinese adult population (≥ 45 years) that IR mediates approximately 11.78% of the total effect on low muscle mass. Accounting for 40%–50% of adult lean mass, skeletal muscle is a tissue sensitive to insulin [31–33] and the main location for glucose uptake mediated by insulin. This positions muscle mass as a critical determinant of systemic IR [28]. Notably, IR is made worse by age‐related muscle mass loss, creating a bidirectional pathophysiological loop. Consequently, the TyG index could be used as a proxy for muscle mass status.

### 4.2. How TyG‐Obesity Composite Indicators Relate to Low Muscle Mass

WC, WHtR, and WWI are established anthropometric measures of adiposity. Among these, WWI is an emerging obesity indicator that appears to outperform body weight and WC in assessing lean mass and fat mass [34]. As a pioneering investigation, this study establishes a link between low muscle mass risk and elevated TyG‐obesity composite indices. Three interconnected mechanistic pathways could account for how obesity, IR, and reduced muscle mass are interconnected, as found in this study. First, the metabaging cycle plays a central role in obesity‐related muscle depletion [35]. The high‐fat milieu characteristic of obesity promotes lipid spillover and ectopic fat accumulation in skeletal muscle, a process known as myosteatosis. The ectopic deposition of lipids within myocytes induces mitochondrial dysfunction, disrupts fatty acid β‐oxidation, and enhances ROS generation. These perturbations culminate in IR and chronic inflammation, key drivers of muscle mass [29, 31]. Second, obesity often coexists with chronic metabolic disorders that perturb muscle protein turnover [36]. Third, lifestyle factors prevalent in obesity, such as sedentary behavior, excessive intake of ultra‐processed food [37, 38], and adherence to proinflammatory dietary patterns [39], further exacerbate muscle mass by independently suppressing protein synthesis and promoting catabolic pathways [36]. In conclusion, prospective research studies are warranted to confirm these results longitudinally and investigate whether IR‐targeted therapies can prevent muscle mass loss in at‐risk populations.

### 4.3. The Current Status of Low Muscle Mass Assessment

The present study reveals that TyG‐WHtR outperforms other TyG‐based obesity indicators in predicting low muscle mass among individuals without diabetes. This advantage likely reflects WHtR’s superior representation of central adiposity, which correlates more robustly with IR and metabolic impairment than either waist circumference or weight‐adjusted metrics. Obesity promotes muscle loss via pathways involving lipotoxicity and systemic inflammation [35], as elaborated in the preceding discussion. Consequently, the TyG index combined with obesity‐related metrics predicts low muscle mass more accurately than the TyG index alone. Notably, the way adipose tissue is distributed regionally represents a stronger determinant of metabolic risk than total fat mass alone. Abdominal obesity more strongly impacts IR and metabolic dysfunction than peripheral adiposity [40], and both are established drivers of muscle loss. Thus, WHtR and WC—as markers of central obesity—exhibit greater accuracy in predicting low muscle mass compared to alternative anthropometric measures. Interestingly, despite also serving as an indicator of abdominal obesity, WWI showed comparatively lower predictive accuracy for low muscle mass. This may be attributed to WWI’s incorporation of total body weight without height adjustment, potentially introducing bias from varying body compositions [41]. Furthermore, although comparable AUC values for TyG‐WHtR across gender and age subgroups were observed, the optimal cutoff points for identifying low muscle mass were found to be subgroup‐specific. The insignificant difference in AUC indicates that TyG‐WHtR is a stable and nearly equivalent diagnostic indicator in different subgroups. However, the observed variation in optimal cutoff values across subgroups underscores the need for threshold selection tailored to specific clinical contexts, rather than a blanket threshold. Muscle mass declines progressively with age, with an estimated annual loss of approximately 1% beginning after the age of 40 years [42–44]. Muscle mass also exhibits sex‐specific differences, with men generally possessing greater muscle mass than women. To achieve consistent diagnostic accuracy across populations with distinct demographic characteristics, cutoff values require population‐specific calibration. Therefore, this study advocates using gender‐ and age‐specific cutoff values for the diagnosis and screening of low muscle mass, rather than “one‐size‐fits‐all.” Implementing subgroup‐specific cutoff values enhances the precision of screening and diagnosis for low muscle mass, thereby facilitating early detection, timely intervention, and ultimately early screening for sarcopenia.

Low muscle mass diagnosis in primary care remains inadequately addressed, owing largely to equipment limitations and practical feasibility concerns. The introduction of TyG‐WHtR offers a promising solution for diagnosing low muscle mass in primary care. This index is user‐friendly, requiring only physical measurements, triglyceride levels, and fasting blood glucose levels, without the need for substantial manpower, material resources, or financial input. It holds potential for future use in diagnosing loss of muscle mass in people without diabetes. However, the present study constitutes an initial investigation into the utility of TyG‐WHtR for diagnosing low muscle mass, and more research is warranted to compare its diagnostic performance against established indicators.

### 4.4. Strengths and Limitations

Our study is distinguished by several key strengths. As the first investigation to examine TyG‐obesity indices in relation to low muscle mass, this study offers a clinically accessible and robust tool for risk stratification. Moreover, by incorporating the novel WWI into the TyG index framework, this research establishes an important benchmark for future studies focused on WWI. The incorporation of NHANES, a sizable, population‐representative database, supports our findings even more.

However, a number of limitations deserve attention when drawing conclusions from this research. First, this study examined low muscle mass, not sarcopenia; thus, how TyG‐obesity indices relate to sarcopenia was not evaluated. It is worth emphasizing that low muscle mass constitutes a core criterion for sarcopenia. However, direct assessment of muscle mass remains challenging in primary care settings due to the limited availability of specialized equipment. This study employs the TyG‐obesity indices for the early screening of low muscle mass, providing a solution to address this clinical bottleneck in primary care and holding significant clinical application value. Second, given the potential confounding effects of fat infiltration and body hydration status [45], the muscle mass obtained from DEXA may have a certain degree of systematic error. It should be noted, however, that this approach affords reliable quantification of muscle mass and is widely recognized in sarcopenia guidelines as a valid diagnostic tool [23, 46, 47]. Thus, it is completely feasible to assess muscle mass using this method. Moreover, potential biases arising from missing data within the database cannot be entirely ruled out and may have influenced the results. In addition, the exclusion of participants with diabetes limits how well our findings can be extended to diabetic populations. To improve it, subsequent research should extend these analyses to include individuals with diabetes. Finally, this study’s cross‐sectional methodology prevents it from establishing causal relationships between low muscle mass and the TyG‐obesity composite indices. Thus, population‐based longitudinal studies should be prioritized in upcoming research to more fully elucidate these relationships.

## 5. Conclusions

Our study indicates that the TyG index, TyG‐WC, TyG‐WHtR, and TyG‐WWI correlate with low muscle mass in those free from diabetes. Greater magnitudes of the TyG‐obesity composite indices correlated with progressively rising odds of low muscle mass. Among the indices evaluated, TyG‐WHtR demonstrated superior performance in predicting low muscle mass. However, definitive conclusions regarding the most accurate predictor of low muscle mass await confirmation through additional prospective studies.

## Author Contributions

Conceptualization: Yue Xi and Genqing Xie; methodology: Jiaqi Huo; software: Jiaqi Huo; validation: Yue Xi and Fang Li; formal analysis: Jiaqi Huo; data curation: Ting Wang and Simiao Tang; writing–original draft preparation: Jiaqi Huo and Ting Wang; writing–review and editing: Jiaqi Huo and Simiao Tang; visualization: Fang Li and Simiao Tang; supervision: Genqing Xie and Ting Wang; project administration: Yue Xi; funding acquisition: Genqing Xie.

## Funding

This research was funded by the Department of Science and Technology of Human Province (2024JJ9058), the Scientific Research Launch Project for new employees of the Second Xiangya Hospital of the Central South University (QH20230224), and the Xiangtan Medical Association (2024‐xtyx‐48).

## Disclosure

All authors have read and agreed to the published version of the manuscript.

## Ethics Statement

The authors have nothing to report.

## Consent

The authors have nothing to report.

## Conflicts of Interest

The authors declare no conflicts of interest.

## Supporting Information

Additional supporting information can be found online in the Supporting Information section.

## Supporting information


**Supporting Information** Table S1: Baseline characteristics of the study participants without taking lipid‐lowering drugs. Table S2: Association between the TyG index and its combination with obesity indicators and low muscle mass among US adults without taking lipid‐lowering drugs in NHANES 2011–2018.

## Data Availability

The data that support the findings of this study are openly available in the NCHS at https://www.cdc.gov/nchs/nhanes/.
