# Supplementary material for: The TyG Index and Obesity Indicators Predicting Low Muscle Mass in US Adults Without Diabetes: NHANES 2011–2018
Source: J Nutr Metab. 2026 Jul 13;2026:1195681. doi: 10.1155/jnme/1195681 (PMC13359029; doi:10.1155/jnme/1195681)
Supplement: Supplementary file 1 — Supporting Information Table S1: Baseline characteristics of the study participants without taking lipid‐lowering drugs. Table S2: Association between the TyG index and its combination with obesity indicators and low muscle mass among US adults without taking lipid‐lowering drugs in NHANES 2011–2018. [file JNME-2026-1195681-s001.docx]

**Table S1.** Baseline characteristics of the study participants (n = 2509, n (%))

| **Characteristics** | **Overall**  **n = 2509** | **Low muscle mass** | | ***P*** |
| --- | --- | --- | --- | --- |
|  |  | **Yes**  **n = 607 (24.2)** | **No**  **n = 1902 (75.8)** |  |
| Age, years | 37.2 ± 11.2 | 40.3 ± 11.1 | 36.2 ± 11.0 | **< 0.001** |
| BMI, kg/m^2^ | 27.90 ± 6.09 | 32.80 ± 6.08 | 26.30 ± 5.19 | **< 0.001** |
| Gender, n (%) |  |  |  | 0.941 |
| Female | 1276 (50.9) | 310 (51.1) | 966 (50.8) |  |
| Male | 1233 (49.1) | 297 (48.9) | 936 (49.2) |  |
| Education level, n (%) |  |  |  | **0.004** |
| Middle school or below | 415 (16.5) | 126 (20.8) | 289 (15.2) |  |
| High school | 522 (20.8) | 120 (19.8) | 402 (21.1) |  |
| College graduate or above | 1572 (62.7) | 361 (59.5) | 1211 (63.7) |  |
| Marital status, n (%) |  |  |  | **< 0.001** |
| Married | 1185 (47.2) | 317 (52.2) | 868 (45.6) |  |
| Never married | 720 (28.7) | 127 (20.9) | 593 (31.2) |  |
| Other | 604 (24.1) | 163 (26.9) | 441 (23.2) |  |
| PIR, n (%) |  |  |  | **0.009** |
| < 1.30 | 828 (33.0) | 231 (38.1) | 597 (31.4) |  |
| 1.30 ~ 3.50 | 887 (35.4) | 205 (33.8) | 682 (35.9) |  |
| ≥ 3.50 | 794 (31.6) | 171 (28.2) | 623 (32.8) |  |
| Physical activity level, n (%) |  |  |  | **< 0.001** |
| No physical activity | 417 (16.6) | 145 (23.9) | 272 (14.3) |  |
| Low physical activity | 1103 (44.0) | 249 (41.0) | 854 (44.9) |  |
| High physical activity | 989 (39.4) | 213 (35.1) | 776 (40.8) |  |
| Smoke, n (%) |  |  |  | **< 0.001** |
| Yes | 963 (38.4) | 266 (43.8) | 697 (36.6) |  |
| No | 1546 (61.6) | 341 (56.2) | 1205 (63.4) |  |
| Alcohol use, n (%) |  |  |  | 0.794 |
| Yes | 1931 (77.0) | 464 (76.4) | 1467 (77.1) |  |
| No | 578 (23.0) | 143 (23.6) | 435 (22.9) |  |
| Self-reported Cancer, n (%) |  |  |  | **0.008** |
| Yes | 80 (3.2) | 29 (4.8) | 51 (2.7) |  |
| No | 2429 (96.8) | 578 (95.2) | 1851 (97.3) |  |
| Hypertension, n (%) |  |  |  | **< 0.001** |
| Yes | 432 (17.2) | 153 (25.2) | 279 (14.7) |  |
| No | 2077 (82.8) | 454 (74.8) | 1623 (85.3) |  |
| Dietary energy, kcal | 2070.00 ± 703.00 | 2000.00 ± 695.00 | 2090.00 ± 704.00 | **0.005** |
| Dietary protein, gm | 83.20 ± 34.80 | 78.90 ± 32.10 | 84.60 ± 35.50 | **< 0.001** |
| Dietary Vitamin D, mcg | 4.52 ± 4.57 | 4.08 ± 4.02 | 4.67 ± 4.72 | **0.002** |
| TyG index | 8.40 ± 0.61 | 8.61 ± 0.57 | 8.33 ± 0.61 | **< 0.001** |
| TyG-WC | 799.00 ± 158.00 | 934.00 ± 149.00 | 756.00 ± 135.00 | **< 0.001** |
| TyG-WHtR | 4.76 ± 0.94 | 5.61 ± 0.85 | 4.49 ± 0.79 | **< 0.001** |
| TyG-WWI | 90.00 ± 10.30 | 97.70 ± 8.82 | 87.50 ± 9.43 | **< 0.001** |

^Low muscle mass was defined as below the P^_25_ ^of the weight-adjusted appendicular lean mass. Bolding indicates statistically significant values,^ *^P^* ^< 0.05.^

**Table S2.** Association between the TyG index and its combination with obesity indicators and low muscle mass among US adults in NHANES 2011-2018 (OR (95%CI), n = 2509)

|  | Crude Model | | Model Ⅰ | | Model Ⅱ | | | Model Ⅲ | | |
| --- | --- | --- | --- | --- | --- | --- | --- | --- | --- | --- |
|  | Crude OR (95%*CI*) | *P* | Adjusted OR (95%*CI*) | *P* | Adjusted OR (95%*CI*) | | *P* | Adjusted OR (95%*CI*) | | *P* |
| TyG index | 2.14  (1.72, 2.66) | **< 0.001** | 1.93  (1.52, 2.46) | **< 0.001** | 1.92  (1.50, 2.46) | | **< 0.001** | 1.83  (1.42, 2.36) | | 0.110 |
| Q1 | Reference |  | Reference |  | Reference | |  | Reference | |  |
| Q2 | 2.27  (1.61, 3.20) | **< 0.001** | 1.97  (1.39, 2.80) | **< 0.001** | 1.93  (1.34, 2.78) | | **< 0.001** | 1.88  (1.28, 2.75) | | **0.002** |
| *P* for trend | **< 0.001** |  | **< 0.001** |  | **< 0.001** | |  | **0.002** | |  |
| TyG-WC | 4.22  (3.52, 5.06) | **< 0.001** | 4.13  (3.46, 4.93) | **< 0.001** | 4.18  (3.49, 5.02) | | **< 0.001** | 4.12  (3.43, 4.96) | | **< 0.001** |
| Q1 | Reference |  | Reference |  | Reference | |  | Reference | |  |
| Q2 | 8.97  (6.40, 12.60) | **< 0.001** | 8.32  (5.96, 11.60) | **< 0.001** | 8.42  (6.05, 11.70) | | **< 0.001** | 7.96  (5.72, 11.10) | | **< 0.001** |
| *P* for trend | **< 0.001** |  | **< 0.001** |  | **< 0.001** | |  | **< 0.001** | |  |
| TyG-WHtR | 4.57  (3.83, 5.45) | **< 0.001** | 4.61  (3.82, 5.57) | **< 0.001** | 4.67  (3.85, 5.66) | | **< 0.001** | 4.62  (3.80, 5.62) | | **< 0.001** |
| Q1 | Reference |  | Reference |  | Reference | |  | Reference | |  |
| Q2 | 11.30  (7.99, 15.90) | **< 0.001** | 10.50  (7.34, 15.00) | **< 0.001** | 10.70  (7.53, 15.10) | | **< 0.001** | 10.20  (7.22, 14.40) | | **< 0.001** |
| *P* for trend | **< 0.001** |  | **< 0.001** |  | **< 0.001** | |  | **< 0.001** | |  |
| TyG-WWI | 3.39  (2.83, 4.07) | **< 0.001** | 3.33  (2.78, 3.98) | **< 0.001** | 3.31  (2.77, 3.96) | | **< 0.001** | 3.23  (2.71, 3.86) | | **< 0.001** |
| Q1 | Reference |  | Reference |  | Reference | |  | Reference | |  |
| Q2 | 5.78  (4.19, 7.98) | **< 0.001** | 5.23  (3.80, 7.19) | **< 0.001** | 5.19  (3.77, 7.14) | **< 0.001** | | 4.90  (3.51, 6.86) | **< 0.001** | |
| *P* for trend | **< 0.001** |  | **< 0.001** |  | **< 0.001** |  | | **< 0.001** |  | |

ORs and 95%CIs were calculated with the use of the Logistic regression model. The crude model did not adjust for any covariates. ModelⅠwas adjusted for age, sex, education level, marital status and family income-to-poverty ratio. ModelⅡwas adjusted for ModelⅠ+ dietary energy, protein and vitamin D. Model Ⅲ was adjusted for modelⅡ+ smoking, alcohol use, exercise, self-reported cancer, high blood pressure.
